# AAPM MPLA case: Betrayal versus disillusionment

**DOI:** 10.1002/acm2.14554

**Published:** 2024-12-02

**Authors:** William N. Duggar, Dongxu Wang, Irina Vergalasova, Cassandra Stambaugh, Leonard Kim

**Affiliations:** ^1^ University of MS Medical Center Jackson Mississippi USA; ^2^ Memorial Sloan Kettering Cancer Center Middletown New Jersey USA; ^3^ Rutgers Cancer Institute of New Jersey New Brunswick New Jersey USA; ^4^ Tufts Medical Center Boston Massachusetts USA; ^5^ MD Anderson Cancer Center at Cooper Camden New Jersey USA

**Keywords:** case study, leadership, leading up, MPLA, professionalism

## Abstract

This work of fiction depicts a scenario in which a faculty member felt they were criticized unfairly and inappropriately for honesty on a faculty survey, which reflected poorly on administration. The faculty member was left struggling with how to respond to conflicting feelings and perception of misaligned goals and mission. Simultaneously, the department chair felt they were blindsided by issues that could have been addressed without the embarrassment of a poor survey. The intended use of this case, through group discussion, self‐study, or role‐play, is to encourage readers to discuss the situation at hand, inspire professionalism and leadership thinking, and allow the practice of conflict management. Facilitator's notes are available upon request to the MPLA Cases Subcommittee. This case study falls under the scope of and is supported by the Medical Physics Leadership Academy (MPLA), a committee in the American Association of Physicists in Medicine (AAPM).

## 1 INTRODUCTION AND INSTRUCTIONS FOR EDUCATION USE

This MPLA case S1, with its accompanying discussion questions, describes a heated conversation between a department chair (Dr Jones) and a faculty member (Don) that the chair had trusted. The chair was disappointed and felt betrayed that the faculty submitted negative feedback about the department to an oversight committee without addressing those concerns with the chair first. The faculty member felt betrayed and disillusioned that the chair took such feedback very personally.

This case can be used to train faculty on how to respond to negative experiences or feedback from superiors. Faculty in leadership positions can grow in how to respond to feelings of betrayal or surprise criticisms. If adapted and used in a role‐play format to practice a difficult conversation, the facilitator should only distribute one version of the backstory to the corresponding participant/actor and organize a conversational practice. Suggestions for facilitation can be found in the Appendix.

Name, gender, and other identities (if perceived) in this case are only representative. The facilitator has the discretion to change names and gender to be more representative of their specific context. The sample facilitator's notes, as well as the editable version of the case, are available upon request to the MPLA cases subcommittee (https://www.aapm.org/org/structure/default.asp?committee_code=MPLACA).

### 1.1 Case narrative

Don worked at a small academic medical center and had done so for several years. His department chair, Dr Jones, a radiation oncologist, had been there for much longer, having seen the department move from being nominally academic, staffed mostly by locums to the blooming department it is today with strong presence from physician and physics faculty. During her tenure, physician and physics residencies had started leading to even more complexity. Don had first joined as a physics resident and then stayed on as faculty with both sides deeming it a mutually beneficial arrangement. During his first few years as faculty, he had become somewhat of a confidante for his chair who often sought his feedback about various happenings in the department. Don had developed confidence as faculty and tended not to be shy about opportunities for growth in the department. To some extent, he had even been mentored by his chair to not only offer feedback, but also possible solutions when communicating about problems. However, despite having significant respect for the growth of the department and his chair, Don felt that as a whole the department had reached some level of stagnation. Specifically, he felt that they could do much better with efficiency, specifically accountability in this area, and that despite having the ear of his chair about the issue, he had the impression that little progress had been made. When faculty affairs asked everyone to fill out a survey regarding department leadership, Don decided to be open and honest about his perception that there could be improvements in accountability. In his mind, it was just as important to provide good feedback to faculty affairs as anyone else. Otherwise, what was the point of surveys? (Supporting Information)

#### 1.1.1 The pipe bursts

Don stepped aside from the doorway to allow traffic to flow out of his department chair's office. He could see Janet's face as she walked out and noticed neither his smile nor his eye contact was returned. He couldn't help but notice the strangeness of the encounter as they were normally friendly toward each other. Though being summoned to his department chair's office was common, he had only a moment to consider that this may not be the typical informal meeting between him and his department chair. “Don, have a seat,” invited Dr Jones. Don took a seat in one of the cushioned chairs across from Dr Jones desk. Again, an unsettling feeling continued to develop as Don could tell this was not a social call. “I'm not happy,” explained Dr Jones. Don just let her continue. “The results came back and they weren't good,” continued Dr Jones. “Do you know what is going on?”

Don searched his memory for any idea of what Dr Jones could be talking about. He could not place any results that he was expecting. Dr. Jones must have read his lack of recall off his face and offered, “You know, the faculty survey?” Realization came crashing down on Don. Of course, he remembered the survey now. He had participated in the survey over 3 months ago, as the entire faculty had been strongly encouraged to fill it out. He also remembered discussing the survey with a fellow faculty member who indicated their plans to just choose the rosy answers in order to not stir anything up. Don, however, had decided to be honest about his frustrations with certain aspects of the department and how it could be better. “Otherwise, how else can the department improve if issues are not brought to light?” he had thought to himself back then. Based on Dr Jones’ reaction today, apparently, he had not been the only faculty member to provide honest feedback.

Don acknowledged his memory of the survey and asked “not good in what way?”

Dr Jones clarified, “Apparently several faculty members have indicated that they are currently dissatisfied with the department and its leadership. Specifically, the complaints are that the department doesn't do very well in accountability. I just got out of a meeting with Faculty Affairs over this survey and it was painful! I want to know what is going on.”

Don decided to provide feedback. “Well, we actually have departmental deadlines set for getting patients from simulation to treatment ‘quickly’. However, I generally have the impression that we rely a lot on our faculty members to be self‐motivated and those that aren't perhaps aren't held accountable to meet the standards that we have already set.” Don had often been frustrated that many faculty members often did not appear to make much of an effort to meet patient care deadlines simply due to perceived poor work ethic rather than actual unavoidable delays. Admittedly, in an academic department, there can be complexities with research time and such, but he felt that the set standards allowed for those complexities.

Dr Jones face changed to one of realization. “You are one of the faculty who responded poorly,” she accused.

Don's face flushed. He hadn't thought it would necessarily “give him away,” but he also didn't really think of that as a negative, because he thought that healthy discussion may be the result of the survey when he filled it out. Now, doubts began creeping into his mind.

“Why not come talk to me? Why would you put criticisms like that on a real survey without talking to me about it first?” Dr Jones inquired. “You can't do that to a chair. It's dangerous for them.” “Plus, it is always better to resolve things in the department. Sometimes the water can get muddy when you get too many players involved.”

Now it was Don's turn to come to realization. “Does Dr. Jones care more about her reputation than the fact there may be real issues affecting our patient care?” he thought. “I thought it would be welcome to be honest on this survey, but now I am not sure Dr. Jones and I have the same priorities regarding the department,” his thoughts continued. He processed Dr Jones’ questions. He did not want to directly say what he really felt: that his concerns had been brushed under the rug when he had observed similar issues over the years and come to discuss them in person. He had felt the survey was a chance to place these concerns into a public discussion format so they could finally be addressed.

Don responded to Dr Jones. “I was really just trying to be honest to the questions being asked on the survey. I genuinely have some thoughts on how we can be better as a department and I didn't think being honest about them on the survey would be a big deal.”

“I am not saying you shouldn't have been honest, but only that you should have talked to me first. I have been telling Faculty Affairs how great we are and here comes this survey. It looks terrible. If you had talked to me, we could have discussed and you still could have been honest on the survey. Do you understand?” Dr Jones questioned, exasperated.

Don squirmed in his chair a little. “I guess, but I thought our priority was to offer the best patient care and my goal is always to assess and deal with issues that prevent that, regardless of how hard it is to hear. I promise I don't mean anything personal by it.”

Dr Jones continued her explanation, “This kind of thing can make or break a department chair. It is not trivial to have Faculty Affairs receive this kind of negative feedback from many faculty members. I will be fine, but this kind of criticism should be handled differently.”

Don's thoughts were racing. “I am sorry. I can see how it would not look good to be surprised by this. I guess I just didn't think it would be a surprise. I promise that I mean well for the department and all the faculty, including you.”

Dr Jones dismissed Don with a final statement, “We can keep talking about this going forward, but just try to think about it from my perspective, please. I want the best for the department too.”

### 1.2 Afterthoughts

Later, Dr Jones sat in her favorite chair with her favorite drink at home after work. Her thoughts scanned over the day's events. “How can my faculty feel this way and not tell me directly? I thought we had a good department, and we were doing well. I feel so betrayed.”

Also at home, Don lay in bed processing the experience in Dr Jones’ office. “I can't believe I misread Dr. Jones so badly. I am so disillusioned. I thought she would not only appreciate feedback but be ready to work to fix it. She may care more about her reputation than anything else. I don't know if I can work under a leader with whom I disagree fundamentally. Guess I will be firing up the old CV tomorrow. I know there are other places that will value a faculty member who wants to make things better rather than accept the status quo.”

The Chair of the Medical Physics Leadership Academy Cases Subcommittee (MPLACA) has reviewed the required Conflict of Interest statement on file for each member of MPLACA and determined that disclosure of potential Conflicts of Interest is an adequate management plan.

The members of MPLACA listed below attest that they have no potential Conflicts of Interest related to the subject matter or materials presented in this document.

William N. Duggar, PhD

Dongxu Wang, PhD

Irina Vergalasova, PhD

Cassandra Stambaugh, PhD

Leonard Kim, MusD

## AUTHOR CONTRIBUTIONS

**William N. Duggar**: Initial concept; manuscript drafting; and revisions. **Dongxu Wang**: Concept revision; manuscript drafting and revisions. **Irina Vergalasova**: Manscript revisions; case piloting as facilitator. **Cassandra Stambaugh**: Concept revision; manuscript revisions. **Leondard Kim**: Concept revision; manuscript revisions.

## CONFLICT OF INTEREST STATEMENT

The authors declare no conflicts of interest.

## Supporting information



Supporting Information

## SAMPLE FACILITATOR'S GUIDE OR SELF‐STUDY GUIDE

### Case overview

After working together for many years, a physician department chair and one of the junior faculty members who stayed on as a physicist after residency experience an unexpected conflict. This happens despite the fact that the chair and the faculty have worked together for some time and have developed camaraderie and trust. Due to persisting frustration about continuously not meeting internal patient care deadlines related to patient workflow, the junior faculty responds honestly on a survey with the intention of departmental improvement, but that impacts the reputation of their department chair and perhaps their plans for addressing these issues by involving personnel external to the department. The negative feedback surprises the chair leading to a defensive posture and initiates personal meetings with various faculty. Though face‐to‐face meetings with the chair were not out of the ordinary, the tone and content of the meeting gives the impression to the junior faculty that his desire for department improvement is less important than the chair's reputation. Upon discovering that this junior faculty, whom she trusted, was one of those providing negative feedback, the chair felt betrayed that the topics were not discussed adequately ahead of the survey. The junior faculty member felt disillusioned that their chair's priorities may be more selfish than noble in response to a negative survey.

This case explores the real challenges of mismatched expectations in the workplace, ambiguities in relationships, and optimal communication. Don and Dr Jones began the conversation with mutual respect and perhaps even admiration, but that relationship was injured from both sides. We leave them both before they have had time to fully process their experience and take steps to repair their relationship and re‐align objectives. The value of this case study is to consider how those involved in the conflict can respond in a manner that will foster growth for both the individuals and the department.

**Learning objectives (LO)** 1 The underlined terms are the key curriculum topics of Medical Physics Leadership Academy (MPLA). See https://w3.aapm.org/leadership/curriculum.php. :

1. Mindfulness of Empathy for other perspectives.
2. Develop organizational awareness and examine a clinical faculty's relationship with the department chair, with the institution, and with the patients.
3. Experiences/conflicts like this can impact professional vitality, even leading to burnout. Consider strategies which might prevent this for either party.
4. Discuss ideal teamwork and collaboration.

**Discussion questions**:

1. In what ways could Don or Dr Jones color their perspectives with more empathy for each other as they process the conflict?
2. In what ways does this situation indicate a failure in organizational awareness from those involved and how could that improve? Additionally, what elements of teamwork and collaboration might have been missing?
3. What are strategies to build robustness or resilience of professional vitality to experiences like this? How about to repair injuries to vitality from events like this?

**Sample answers**:

1. Both could begin by considering alternatives to their initial impressions. Empathy is the capacity or ability to imagine oneself in the situation of another. Don could imagine how he would feel if he were the department chair in the situation receiving negative feedback, while Dr Jones may remember similar experiences from her time as faculty under a chair feeling frustrated about lack of change in the face of challenges. Don may perhaps challenge himself to imagine whether Dr Jones might have more noble reasons for her reaction. Don may also reach out to contacts in similar positions of leadership to pose the question to them for a different and objective perspective. Dr Jones may discuss the situation with a coach or mentor. Additionally, Dr Jones and Don could follow‐up in a more relaxed setting to discuss the response further to better understand their perspectives.
  2
  See recommended MPLA curriculum on empathy. AMERICAN ASSOCIATION OF PHYSICISTS IN MEDICINE (aapm.org)
2. Both Don and Dr Jones are at risk of missing the big picture. The conflict arose because both were surprised by new information. As a faculty, understanding the goals, objectives, and motivations of both the organization and leadership are of paramount importance for ensuring effectiveness of daily activities. “Swimming against current” is usually not desirable. As a chair, being in touch with the perspectives, feelings, and status of faculty and staff is essential and requires humility, hunger for honest feedback, and an open ear (not just open door). Better awareness starts with acknowledging current possible ignorance and bias so that new information can be sought and processed optimally.
  3
  See recommended MPLA curriculum on organizational awareness. AMERICAN ASSOCIATION OF PHYSICISTS IN MEDICINE (aapm.org)
   It seems that if feedback culture was in place, it may need structural improvements. For example, Don either did not feel heard or did not perceive progress or improvement efforts and Dr Jones was defensive in the face of more than one faculty member. Consistent communication may improve that perception. Initial defensive posture can also inhibit progress. Feedback should not only be sought but also assigned value even when negative. Don also may have expressed his frustrations without offering solutions or perhaps have been impatient with how long progress can take. Approaching issues with patience and potential solutions can help with mitigating defensive attitudes/responses. Had Don been as direct in previous communication as he had in the survey that he was so dissatisfied with the department? Was Dr Jones really listening to the feedback she was given either via survey or in person? Often, we remember ourselves in the best light. Ascribing good intentions to each other could also help with the ability to collaborate even in light of misunderstandings.
  4
  See MPLA curriculum on teamwork and collaboration. AMERICAN ASSOCIATION OF PHYSICISTS IN MEDICINE (aapm.org)
   One also may wonder how well the department has defined its mission, vision, or values which can be used to build alignment and teamwork. Organizations lacking teamwork or healthy culture may seek to revitalize its collective mission, vision, and values.
3. It takes effort to build robust resilience and professional vitality, and to recover from negative professional events. A few possible strategies could help. One method is simply to take some time off, if possible. By taking the time off, and therefore the mind off this recent event, even for a short period of time, it can help one re‐focus and stop dwelling on the single event. Another approach is to seek professional help, including from a career coach or from a counseling service. These professionals can help uncover one's internal thinking process, and also offer perspectives unseen by oneself. Finally, if an organization has defined its mission/vision/values and one is concerned about missional alignment among the team, it is useful to revisit the organizational mission and personal missions. Often, it can be discovered that the personal and organizational missions align in the big picture; individual setbacks or compromises may only exist in the means but not the ends.
  5
  See recommended MPLA curriculum on professional vitality. AMERICAN ASSOCIATION OF PHYSICISTS IN MEDICINE (aapm.org)
   Organizations without a mission, vision, or values may suffer for it.
  a. Optional activity for value of mission statement (uses the U.S.A. flag as an object lesson, but any flag could be used with some modification to the questions below):
  i. Ask each person to answer these six questions without guidance:
    1. Red or green?
    2. Purple or blue?
    3. Stars or planets
    4. Up in the air or down on the ground?
    5. Metal or cloth?
    6. 5 or 50?
  ii. Show picture of United States of America flag then repeat answering questions together
  iii. Note how alignment improves when something is guiding the answers rather than random preferences
  b. Optional activity: Have each person write three to five sentences describing what drives them or is their source of passion. Suggest the use of that mission statement in an easy reminder location such as on a mirror or saved in their phone. Additionally, writing a personal vision for future goals as well as important personal values can be included in this activity.

