# Supplementary material for: AAPM MPLA case: Betrayal versus disillusionment
Source: J Appl Clin Med Phys. 2024 Dec 2;26(1):e14554. doi: 10.1002/acm2.14554 (PMC11714130; doi:10.1002/acm2.14554)
Supplement: Supplementary file 1 — Supporting Information [file ACM2-26-e14554-s001.pdf]

FORM TO BE COMPLETED BY GROUP AND SUBMITTED TO EXCOM

(Form approved by EXCOM April 28, 2022; minor revision July 22, 2022)

AAPM Group #: MPLACA

Title: Case Study – Betrayed or Disillusioned?

Chair & Vice Chair: Leonard Kim, Cassandra Stambaugh

Other Authors: William N. Duggar, Dongxu Wang, Irina Vergalasova

**Review History**

| | Body | Lead reviewer | Other reviewer(s)* |
|---|---|---|---|
| **council** | PC | Sonja Dieterich | |
| **committee** | MPLAWG | Erica K., Samantha S. | |
| **subcommittee** | | | |
| **work group** | | | |

(*) Additional reviewer names are in the compiled Excel review file history, if needed.

The authors are requesting submission to:

- ○ *Medical Physics*
- ◉ *Journal of Applied Clinical Medical Physics*
- ○ other – Science Council (or other Council) permission required

Are you requesting endorsement requested from additional professional organizations?

- ○ Yes
- ◉ No

If yes, please list organizations:

- ☐ ABR ☐ ABS ☐ ASTRO
- ☐ EFOMP ☐ ESTRO ☐ SDAMPP ☐ Other

Any additional clarification for the parent committees and parent council:

REMINDER for lead reviewer: Sort the order of comments in the attached Excel review file so that the most substantial comments made during prior reviews are listed first, especially consider areas of contradictory comments.

**Items for EXCOM review – to be completed by Council/group and returned to EXCOM**

EXCOM has developed a streamlined approach for the review and approval of the many documents and materials that come to EXCOM for consideration. The number of these documents and the length and detailed nature of many of them make it infeasible for EXCOM to do line-by-line review and copy editing. Nonetheless, as part of the due diligence of AAPM, a type of high-level review is appropriate before these materials are considered approved by AAPM.

EXCOM has developed a strategy that consists of a high-level review of four primary concerns: appropriateness, completeness of domain-expert review, a field safeguard test, and an organizational safeguard test. These concerns are considered by EXCOM using a four rubric question checklist. The Council/group assists EXCOM in this review by filling in the domain-expert review section listed above and two of the four rubric questions, listed below:

1) Field Safeguard Test: During the review of the document/material under consideration, were there concerns related to the professional advancement and recognition of medical physicists and the scope of practice of medical physics? If yes, how were they resolved.
   a) ◯ Yes ◯ No ◯ Uncertain ◯ Not applicable
   b) Details, if applicable:

2) Organizational Safeguard Test:
   a) Does this document/material pose a risk to the AAPM as an organization (e.g., strategic, financial, compliance, operational, reputational)?
      ◯ Yes ◯ No ◯ Uncertain ◯ Not applicable

   b) If yes, have those risks been sufficiently considered and mitigated, where possible?
      ◯ Yes ◯ No ◯ Uncertain ◯ Not applicable

3) Where appropriate, have the interests of other relevant organizations with whom AAPM has relationships been considered?
   ◯ Yes ◯ No ◯ Uncertain ◯ Not applicable

   a) Details, if applicable:

Additional details regarding any of the items above can be entered here as free text (enter name of individual filling out the form and specify if on behalf of the Council or another group):
